# Effect of vitamin D3-fortified fruit juice supplementation of 4000 IU daily on the recovery of iron status in childbearing-aged women with marginally low iron stores: Protocol for an 8-week, parallel group, double-blind randomized controlled trial

**DOI:** 10.1371/journal.pone.0265772

**Published:** 2022-03-25

**Authors:** Salma Faeza Ahmad Fuzi, Loh Su Peng, Nurzalinda Zabaha Zalbahar, Norhafizah Ab. Manan, Muhammad Najib Mohamad Alwi

**Affiliations:** 1 Department of Nutrition, Faculty of Medicine and Health Sciences, Universiti Putra Malaysia, Serdang, Selangor, Malaysia; 2 Department of Public Health, Faculty of Medicine, Cyberjaya University College of Medical Sciences, Cyberjaya, Selangor, Malaysia; 3 International Medical School, Management and Science University (MSU), Shah Alam, Selangor, Malaysia; University of Ottawa, CANADA

## Abstract

**Background:**

In recent years, emerging evidence has highlighted the role of vitamin D as an iron absorption enhancer by suppressing hepcidin concentration, albeit with an unclear underlying mechanism. Dietary-based approach in improving iron status has been widely practised, however, there is a scarcity in randomized controlled trials (RCT) to elucidate the effect of vitamin D-fortified juice on iron status recovery. Therefore, this study aims to investigate the effect of an 8-week vitamin D3-fortified fruit juice supplementation on iron status indicators in childbearing-aged women with marginally low iron stores.

**Methods:**

In a placebo-controlled, double-blind, RCT, a total of 120 women aged between 19–40 with serum ferritin < 20 μg/l and fulfilled the eligibility criteria will be randomized into consuming either vitamin D3-fortified fruit juices containing 4000 IU (100 mcg) (vitamin D) or placebo-fruit juices (placebo) daily for eight weeks. At every 4-week interval, 10 ml fasting blood sample, information on dietary habit and anthropometric measurement will be collected. A mixed model repeated-measures analysis of variance will be performed to determine the effect of the intervention and the interaction with time points for all iron and vitamin D status blood biomarkers.

**Discussion:**

Vitamin D supplementation in food fortification as a novel iron absorption enhancer might be a future and relevant alternative management of iron deficiency as opposed to the oral iron therapy that has poor adherence.

**Trial registration:**

Clinicaltrials.gov: registration number NCT04618289, registration date October 28, 2020, protocol ID JKEUPM-2020-033.

## Introduction

Anemia is one of the most common micronutrient deficiencies, affecting populations worldwide [1]. Half of the time (50%), anemia cases were caused by iron deficiency, leading to multiple negative impacts on human health. A cohesive approach is required to combat this condition since its occurrence is suggested to be multifactorial. Iron supplements have been widely used in at-risk groups, but the efficacy is limited due to its adverse effects, causing low adherence, leading to inefficiency of the intervention [2] and its low bioavailability [3]. Extensive literature, including scientific evidence from systematic reviews, is also available on the effect of dietary-based approach on the improvement of iron status, reporting inconsistent findings [4–11]. Altering individuals’ food preference has been one of the barriers to improving iron status through dietary modification [12], and iron fortification did not completely improved general iron status [13]. A clearer understanding of the interaction between inflammation, erythropoiesis, and hypoxia regulation by the iron regulator peptide hormone, hepcidin, may be useful in designing effective iron interventions that result in fewer adverse effect with maximum advantage [13]. In reference to this, recent studies have presented evidence of the potential utilization of vitamin D as an iron absorption enhancer that suppresses hepcidin, a main iron regulator [14–16]. Besides, observational studies carried out in various settings and populations have suggested an inverse association between increased anemia risk and serum/plasma 25(OH)D concentrations [17].

Despite anemia and vitamin D deficiency being two of the most widespread nutritional problems affecting different age groups across populations, the mechanism that linked these deficiencies remain unclear. There is a scarcity of evidence that supports the links between these deficiencies in the normal population, as previous studies were predominantly carried out in hospital patients [18]. Moreover, it has been suggested that vitamin D plays a role in the erythropoiesis process by decreasing the expression of pro-inflammatory cytokines, which cause the suppression of hepcidin. Consequently, decreased cytokines and suppressed hepcidin leads to higher iron bioavailability for red blood cell (RBC) production and hemoglobin synthesis [19, 20]. It was observed in the in vitro part of a study by Bacchetta, Zaritsky [16] that hepcidin expression was significantly reduced by at least 0.4-fold when treated with 25(OH)D and 1,25(OH)D, whilst Zughaier, Alvarez [20] showed that hepcidin expression was significantly suppressed to between 5 and 10-fold with lipopolysaccharides (LPS) and different concentrations of calcitriol, suggesting direct inhibition of vitamin D metabolites on the transcription of the HAMP gene. A significant reduction of serum hepcidin concentration to 34% and 33% following 24- and 72-hours supplementation of vitamin D2 (100 000 IU) in 7 healthy participants, respectively, was observed in the Bacchetta’s study supporting the in vitro findings, and a moderate but significant negative association between changes in serum hepcidin and 25(OH)D concentrations (r = -0.38, p = 0.02) were observed in 38 kidney patients randomized to receive vitamin D3 (50 000 IU/week), and every other week for 40 weeks or placebo in Zughaier’s.

At present, there is a lack of RCT investigating the effect of vitamin D supplementation using fruit juice as a food fortification vehicle, aiding as an iron absorption enhancer administered routinely, especially in the general population who are at risk of iron deficiency not only in Malaysia but worldwide. Therefore, the present study is designed to determine the effect of an 8-week vitamin D3-fortified fruit juice supplementation on hematological indicators and hepcidin response in a cohort of child-bearing aged Malaysian women with low iron stores, in addition to their habitual dietary intake of iron. In addition to investigating its efficacy, this study also is aimed to assess the effect of a higher dose of vitamin D3 in the fortified fruit juice (4000 IU/day) on iron metabolism. The European Food Safety Authority (EFSA) has set the no-observed-adverse-effect level (NOAEL) of 10 000 IU/day and upper level (UL) of 4000 IU/day for vitamin D, similar to the UL recommendation by the Institute of Medicine (IOM), which is the proposed dose in this study. It was also agreed by the Committee on the toxicity of chemicals in food, consumer products and the environment (COT) that a UL of 4000 IU is appropriate for adults ≥ 18 years and above [21]. It is hypothesized that there will be a significant improvement in hematological indicators following an 8-week daily vitamin D3-fortified fruit juice supplementation in the vitamin D group compared to the placebo group. Furthermore, it is predicted that plasma hepcidin concentration will be reduced, resulting in increased iron stores. Due to the suppressed plasma hepcidin, it is also expected that plasma 25(OH)D concentration will be increased in the vitamin D group as opposed to control group.

## Methods

### Study design and site

The study is a parallel-group, placebo-controlled, double-blind, randomized trial designed to investigate the effect of an 8-week vitamin D3-fortified fruit juice supplementation on hematological indicators and hepcidin response in a cohort of marginally low iron stores, childbearing-aged Malaysian women. The study was registered with the Malaysia National Medical Research Register (NMRR-19-3852-51427), clinicaltrials.gov (NCT04618289), and ethical approval for the study protocol has been obtained from the Universiti Putra Malaysia Ethics Committee for Research Involving Human Subject (JKEUPM) on the 8^th^ May 2020 (JKEUPM-2020-033). The study will be conducted for eight weeks in Selangor, Malaysia, and the data collection will be carried out between July 2021 and July 2022. Participants that will be recruited are childbearing-aged working women residing in urban areas of Serdang, Kajang, Bangi or the vicinity of Selangor state. Potential participants will be recruited from either schools, universities, residential areas or government officials in the specified areas where media such as posters, flyers, emails will be sent to various departments within universities or government officials, discussion boards near residential areas, social media pages and university/government officials’ webpage/intranet.

### Study participants

This study’s inclusion criteria are healthy women of child-bearing age (19–40) and not pregnant or lactating. Candidates with a history of gastrointestinal disorder (celiac disease, Crohn’s disease, irritable bowel syndrome, gastroesophageal reflux disease, peptic ulcers and other related gastrointestinal disorders which may cause nutrient malabsorption) and iron metabolic disorders such as iron overload, had donated blood since the past six months, and regularly consuming nutritional supplements (iron, vitamin D, vitamin C, calcium) will be excluded. Moreover, potential participants must fulfill the screening eligibility criteria: marginally low iron stores and non-anemic.

### Randomization and blinding of participants

The randomization process will be conducted using computer-generated software (www.randomizer.org) by an independent third party. Participants (n = 120) were randomized using permuted block randomization into two groups: vitamin D3-fortified fruit juice (vitamin D group) or placebo-fruit juice (placebo group) in a 1:1 ratio. According to the generated plan, the third party will allocate 62 sachets of vitamin D3-fortified fruit and placebo-fruit juice powder into each supplement container. Each supplement container will then be sealed in a tamper-proof supplement container and numbered before distributed to the participants. Both participants and researcher are double blinded to the group assignment. The numbered supplement will be distributed sequentially to the participants based on the sequence of attendance during their baseline clinic session (Week 0). The blinding will be maintained throughout the study, and allocation will not be unlocked until the end of the data analysis or any adverse event such as hypercalcemia.

### Power and sample size estimation

The sample size was estimated by using (mean ± S.D) serum ferritin concentrations (ug/dl) from a randomized trial carried out by Blanco-Rojo, Pérez-Granados [7] in 41 women aged 18–35 years with low iron stores to determine the effect of iron-fortified fruit juice consumption on iron and vitamin D status. At week 8, it was observed that (mean ± S.D) serum ferritin concentrations were significantly higher in the vitamin D group (34.1 ± 14.8 ug/l), compared to 24.8 ± 17.7 ug/l in the placebo group (p < 0.05). In this study, the effect size is calculated by dividing the mean difference (9.3 ng/ml) with the pooled standard deviation (16.29). With an effect size of 0.571, the total sample size will be required in the present study is 50 individuals/group (Power = 0.80, α err prob = 0.05). By considering a 20% drop-out rate, the total sample size required to demonstrate a significant difference in serum ferritin concentrations between the vitamin D group and placebo is estimated to be 120 (60 participants/group). The sample size was estimated using G-Power Software (Version 3.1.7).

### Intervention

#### Vitamin D3-fortified fruit juice supplement and placebo

The vitamin D3-fortified fruit juice powder contains vitamin D3 cholecalciferol (4000 IU, 100 mcg) and packaged in individual sachets (7 g). Both vitamin D3-fortified fruit juice powder and placebo were custom-produced by Fiatec Biosystem Sdn Bhd, Selangor, Malaysia. Matching placebo were produced in the same manner, without the active ingredients by the same company and similar with the intervention powder in terms of appearance, size, colour, and taste to achieve the double-blind design. A sachet of the intervention powder consists of powdered vitamin D3, permitted sweetener Suitena^TM^ containing Xylitol, Erythritol & Stevia, citric acid, anti-caking agent and similar ingredients used to produce placebo juice without the active ingredient of vitamin D3. In addition, a nutritional analysis in a certified laboratory provided the nutritional composition of the vitamin D3-fortified fruit juice powder: 27 kcal energy, 6.8 g carbohydrate, 0.3 g sugars, 7 mg vitamin C, and 0.3 mg iron per sachet.

All vitamin D3-fortified fruit juice powder supplements are packaged in individual sachet for daily consumption and produced as per the research requirement and according to the Good Manufacturing Practice (GMP) standards. For safety purposes, the fruit juice powder was sampled and sent for microbiological testing and heavy metal analysis to be deemed safe for human consumption. These tests were carried out before the powder is packaged into sachets and in the finished products. None of the production cost of both vitamin D3-fortified and placebo-fruit juice powder was financed by the manufacturers. Participants will be provided with an 8-week supply of the fruit juices, depending on the group assigned during the first baseline visit (Week 0). The researcher will demonstrate the fruit juice preparation methods to ensure the use of a standardized method. Additionally, a standard size measuring cup will be provided for all participants, with written instruction on preparing and consuming the fruit juices at home. If participants failed to consume the juice per instruction in any event, it was advised that they do so at once or later in the day and record it in the provided food diary.

#### Adherence to intervention

Participants will be required to consume 56 sachets of vitamin D3-fortified fruit juice or placebo-fruit juice for eight weeks. However, six extra sachets will be provided in each container (n = 62) for researchers to confirm their adherence once the study was completed. Besides, the participants must retain and hand over all the used sachets consumed between week 0 to 4 during the interim clinic session and all the used and unused sachets consumed from week 4 to 8 during the post-intervention clinic to assess the adherence. Adherence to the intervention (%) will be calculated using the following formula: (62 –remaining sachets in the pot upon return)/56 x100. On top of that, participants must record their fruit juice consumption, missed dose and time (if any) as a mean of assessing adherence in the diary provided. Furthermore, participants are also advised to report any adverse effects related to consumption of the fruit juices during each clinic session and communicate with the researcher concerning adverse events they experienced throughout the study.

### Study visits and procedures

**Fig 1:**
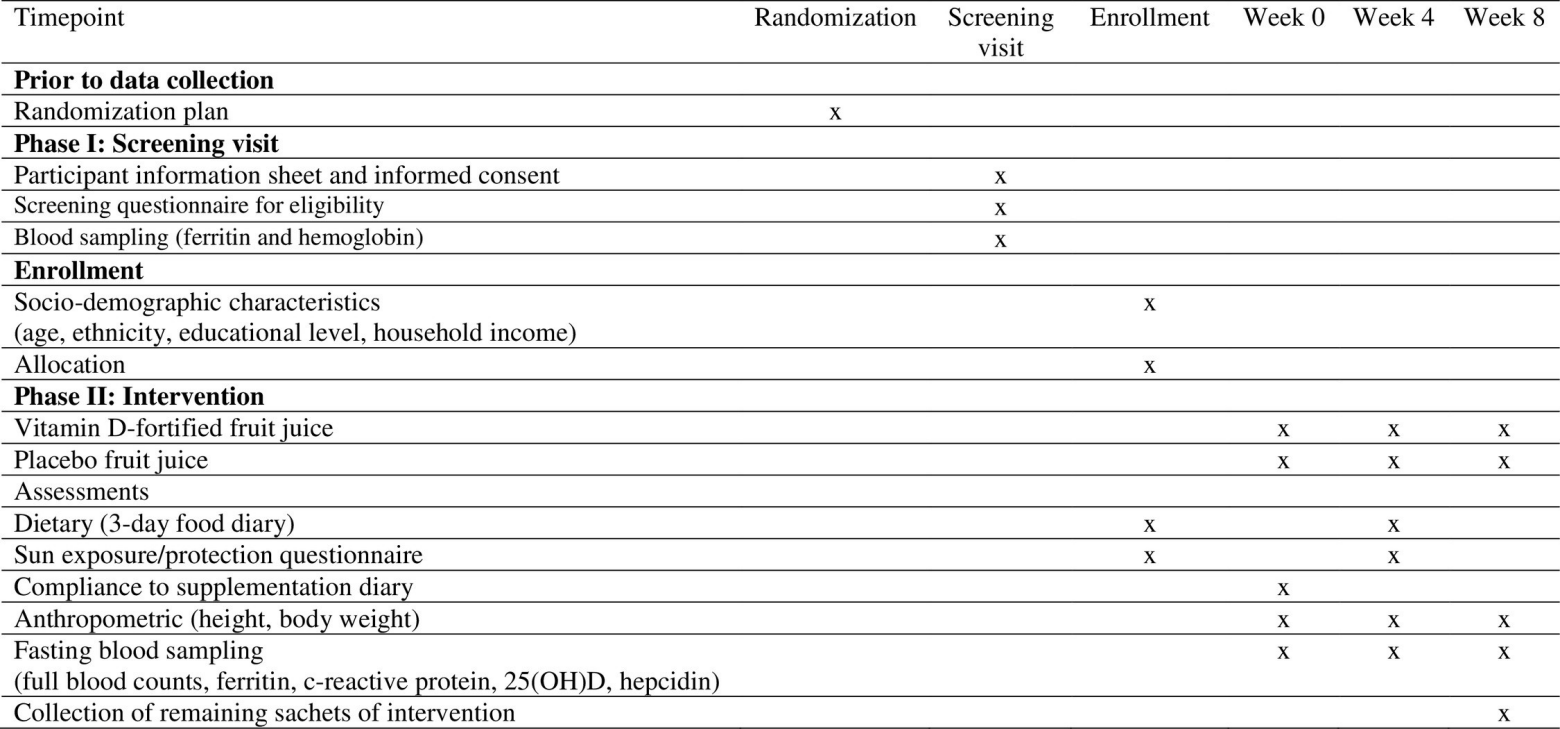
Timepoint of study enrollment, interventions and assessments.

Upon recruitment, they were subjected to a screening process to determine their plasma ferritin and hemoglobin concentrations to include women with marginally low iron stores [plasma ferritin concentration < 20 μg/l] [22, 23] and non-anemic [hemoglobin concentration > 12 g/dl] [24]. **Fig 1** outlines the timepoint of enrollment, interventions, and assessments of study, which included two main phases. Phase I involved the recruitment and screening phase, where potential participants with marginally low iron stores will be identified and randomized. After that, Phase II or the intervention phase will be conducted where all the eligible participants will be supplemented with either vitamin D3-fortified fruit juice containing 4000 IU (100 mcg) (vitamin D group) or placebo-fruit juice (placebo group) daily for eight weeks.

#### Phase 1: Recruitment and screening

The prevalence of iron deficiency based on plasma ferritin concentration (< 15 μg/l) in women aged 18–40 years in Malaysia was reported to be approximately 33% [25]. Meanwhile, the prevalence of adult women who consumed vitamin and mineral supplements based on the Malaysian Nutrition Adult Survey (MANS) 2014 was reported to be approximately 32.1% [26]. Considering these data, approximately 736 women would have to be screened to achieve the required sample size. All participants will be provided with a participant information sheet (PIS) and a date for the venous blood collection to ascertain their plasma ferritin and hemoglobin concentrations, to determine their iron stores and hemoglobin levels. Then, they will be provided with a written consent form and will be required to complete a screening questionnaire to ensure the eligibility criteria are met. The screening questionnaire will be used to obtain the participants’ socio-demographic characteristics, including age, ethnicity, religion, educational level, and household income.

During the screening session, 1 ml of venous blood will be collected at the clinical laboratory at the Department of Nutrition, UPM by a trained phlebotomist. The whole blood and plasma sample collected will be used to determine the concentrations of ferritin and hemoglobin. Once the blood analysis results are obtained, participants will be notified via email or phone. Eligible participants with marginally low iron stores and non-anemic will be invited to participate in Phase 2 of the study. On the other hand, those with abnormally high plasma ferritin concentrations (> 150 ug/l) and low hemoglobin concentrations (< 12 g/dl) from the screening will be advised to arrange a check-up with their registered general practitioner and excluded from the study.

#### Phase 2: Intervention

A total of 120 eligible participants were randomized to receive either 4000 IU (100 mcg) of vitamin D3-fortified fruit juice or placebo-fruit juice. Depending on the assigned group, the participants will be instructed to consume either vitamin D3-fortified fruit juice or placebo-fruit juice daily in the morning by diluting the powder in 150 ml of water for eight weeks. Additionally, participants will be reminded to maintain their dietary habits and physical activity, besides abstaining from donating blood during the study, which may interfere with the interpretation of study findings.

#### Clinic follow-up visits

**Fig 2:**
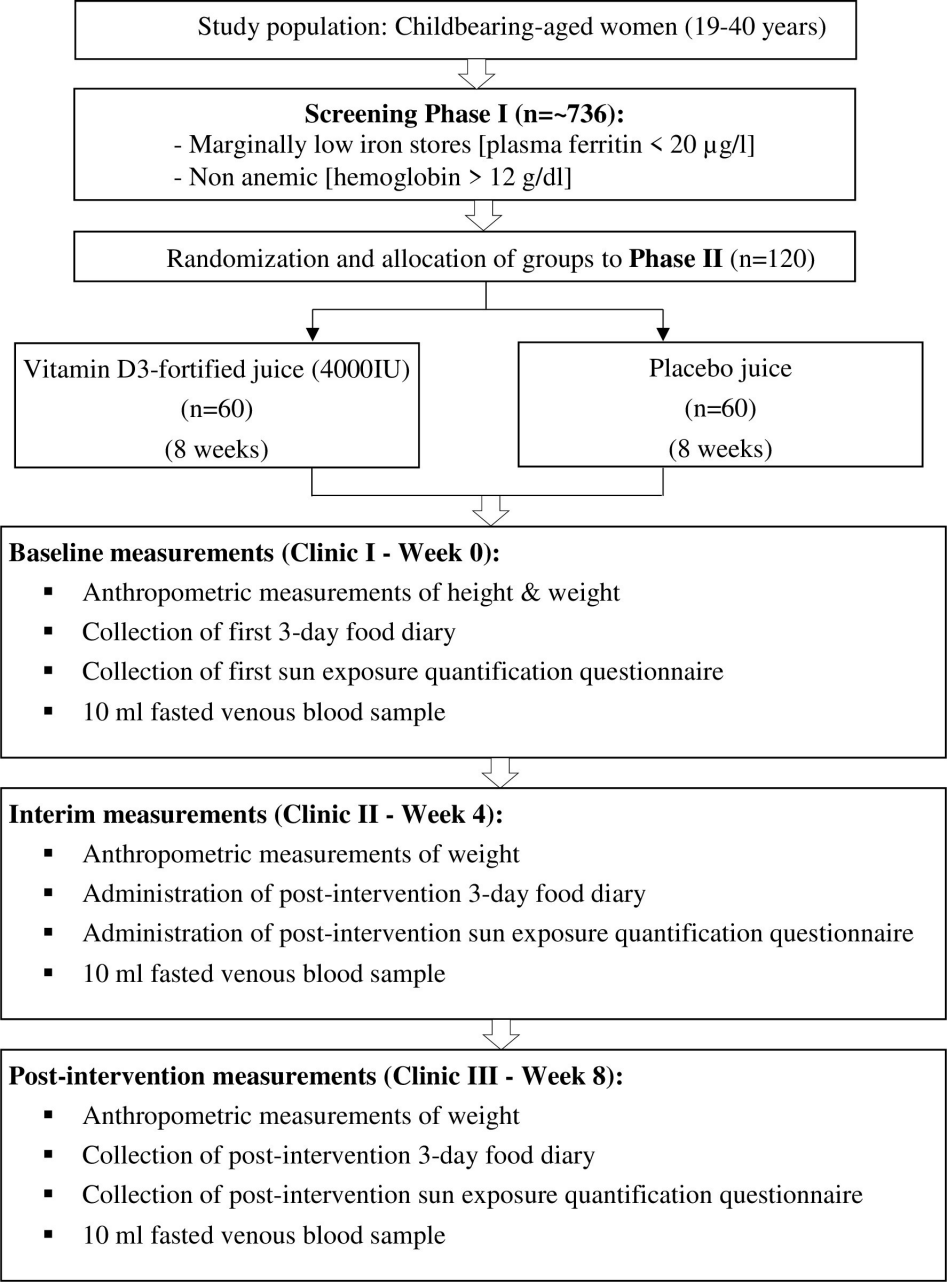
Study protocol overview.

Participants will be required to attend three clinic visits after fasting overnight (± 8 hours) throughout the study at the Department of Nutrition’s clinical laboratory, UPM, where the initial screening session will be carried out. Participants were only allowed to consume only water during the overnight fast. Each session will span approximately 30 minutes, between 8–10 am. The details of each clinic sessions are as follows **(Fig 2)**:

Clinic 1 (Week 0, baseline): Participants’ height (cm) will be measured by using a digital stadiometer and weight (kg) using weighing scales, followed by the collection of a 10 ml fasting venous blood sample. Then, they will be given a 3-day food diary to be completed at home before the clinic meeting and collected during the clinic session. An 8-week supply supplement container containing fruit juice powder in sachets with assigned participant ID will be provided to them. At the same time, the participants will be briefed on the protocol, and an email will be sent one day prior to the following clinic appointment as a reminder.

Clinic 2 (Week 4, interim): The same procedure will be repeated except for height measurement. Participants will be given a 3-day food diary to be completed before the next clinic.

Clinic 3 (Week 8, post-intervention): The same procedure will be repeated except for height measurement. Later, the final 3-day food diary and supplement container (with any) of the remaining fruit juice powder in sachets will be collected to calculate the adherence.

#### Venepuncture procedure and blood sampling

Venepuncture will be performed by a trained phlebotomist (supervised by a medical doctor who is a research team member) to obtain 10 ml of venous blood samples from participants at each clinic session. Both whole blood and plasma obtained from the venepuncture will be utilized in the study analysis. Blood samples were collected in lithium heparin and EDTA blood collection tubes (BD Company, New Jersey, USA) for the various blood biomarker analysis. Whole blood samples will be analyzed immediately after collection to measure full blood counts (FBC). On the other hand, venous blood samples will be centrifuged for 10 minutes (1600 g) at 4°C to obtain plasma samples required for iron and vitamin D biomarkers detection, which will then be aliquot into microcentrifuge tubes and stored at -80°C before analysis. Besides, plasma samples collected at three time-points (baseline, interim and post-intervention) will be used to analyze iron status biomarkers (C-reactive protein, ferritin and hepcidin) and vitamin D metabolism biomarkers (25-OH D) concentrations using commercially available ELISA assay kits specific to different biomarkers.

### Study outcomes and measures

#### Primary outcomes

Blood samples will be collected at three time points during the 8-week course of the study to determine iron status changes and vitamin D biomarkers. The study’s primary outcome will be ferritin concentration, which has been demonstrated to be well-associated with iron storage in the absence of inflammation [27]. A higher concentration of ferritin may be observed with inflammation despite low iron stores, causing the interpretation to be more complex [27]. Nonetheless, this situation can be controlled by measuring the c-reactive protein (CRP), which is included as part of the biochemical analyses in this study. Meanwhile, the full blood count indices (FBC) will provide the amount of functional iron utilized to produce red blood cells [28]. Besides, a peptide hormone that functions to downregulate iron absorption known as hepcidin [29] is also measured to investigate its mechanistic role in iron metabolism homeostasis following vitamin D administration. Plasma 25(OH)D concentration will be measured to determine the circulating vitamin D.

FBC profiling will be carried out using an automated Beckman Coulter Ac.T diff Hematology Analyzer (Bechman Coulter Inc California, USA) and compared to the normal values/ranges [30]. Reagents used to include Coulter reagents and Coulter rinse shutdown diluent will be obtained from Beckman Coulter Inc, California, USA. Besides, commercially available ELISA assay kits will be utilized to determine the iron and vitamin D biomarkers status by measuring the plasma C-reactive protein (CRP) (R&D Systems Inc, Minneapolis, USA), ferritin (Elabscience Biotechnology Co. Ltd), and hepcidin (R&D Systems Inc, Minneapolis, USA) concentrations for iron biomarkers status. Meanwhile, 25(OH)D (Calbiotech Inc, California, USA) concentration will be determined for vitamin D biomarker status. Readings from the concentrations will be compared to the normal thresholds of the specified blood biomarkers [24, 27, 31, 32]. Each ELISA kit assay will be purchased with quality controls (low, medium, high), including with expected range from similar companies supplying the specified kits. A similar assay for each biomarker will be used for all blood samples collected to account for inter-assay coefficient variations.

#### Other measures

Participants’ height will be measured to the nearest 0.1 cm with a wall-mounted digital stadiometer (Model 264 SECA, Hamburg, Germany), whereas their body weight will be recorded to the nearest 0.1 kg using an electronic scale (Model 875 SECA, Hamburg, Germany). The measurements will be obtained in compliance with the standardized methods and protocols by the International Society for the Advancement of Kinanthropometry (ISAK) in duplicates, while the instruments used will be calibrated to ensure accuracy. After that, the body mass index (BMI) will be calculated using the participants’ height and weight and compared to the cut-off point set by the World Health Organisation [33].

Participants will be required to complete a 3-day food diary at baseline and post-intervention (to include 2 weekdays and 1 weekend day) to estimate their habitual dietary intake. The diary comprised of instructions on how to record participants’ dietary intakes properly, including a guide to portion sizes, how to describe the foods/drinks in detail, together with a sample diary. In each section of the diary, columns for the time of consumption, location, description of food/drink consumed, brand and amount/quantity, physical activity, and recipe sections will be provided for the participants to complete. On top of that, they will be given another diary to complete and returned to the baseline and post-intervention clinic. Upon completion, their dietary records will be analyzed for nutritional content by using Nutritionist Pro Software (Axxya System LLC, Washington, USA), and the food items used for the analysis will be based on the Malaysian Food Composition Table [34]. The Malaysian Recommended Nutrient Intake (RNI) 2017 will be used as a reference to compare the participants’ energy, macro, and micronutrients intake [35].

A set of questions will be used to account for sun exposure which may confound study findings. Participants will be asked about their weekly duration under direct sunlight (minutes/day) to quantify sun exposure. Then, the scores will be calculated by multiplying the total daily exposure by the number of days in a specified week. Other than that, participants were required to detail their clothing style for sun protection level, including an umbrella, hat/cap, skin protection lotion, clothes such as long sleeves/pants or skirts and veils. In the end, the sun protection level will be determined by computing the total usage of the eight specified items with a max score of 8 (min = 0) [36]. Questions will be administered before the baseline clinic and during post-intervention clinic sessions.

### Data analysis

All statistical analyses will be performed using IBM SPSS Statistic Data Editor Software (Version 21). Kolmogorov-Smirnov tests and histogram will be used to determine the normality of the data, while the descriptive statistics will be used to describe the frequencies and median (range) or mean (standard deviation) for categorical and continuous data, respectively. Meanwhile, baseline and clinical characteristic comparisons between the groups (vitamin D and placebo groups) will be carried out using independent t-test or Mann-Whitney test for continuous data, and Pearson Chi-square or Fisher’s exact test for categorical data. Mean change from baseline to all follow time points of the primary outcomes (plasma ferritin, full blood counts, C-reactive protein, hepcidin and 25(OH)D) will be compared between treatment groups using repeated-measures analysis of variance (ANOVA). Additionally, treatment group, baseline characteristics, sun exposure and dietary intake will be used as fixed factor or covariates in the analysis. The adjusted mean difference with 95% confidence interval and Cohen d will represent the magnitude of the vitamin D effect. A 5% level of significance will be used to determine statistical significance in all analyses. Finally, treatment effects will be assessed using an intention-to-treat strategy. After that, the sensitivity analysis will be conducted with the last observation carried forward assumption for missing data for the primary outcome.

### Ethics and dissemination

The study will be carried out according to the guidelines in the Declaration of Helsinki. All procedures involving human subjects were approved by the UPM Ethics Committee for Research Involving Human Subjects (JKEUPM reference: JKEUPM-2020-033). Participants’ information sheet with a written consent form was obtained, and later it will be explained to them that they are free to withdraw at any time, without excuse. The study protocol was registered and is publicly accessible at ClinicalTrials.gov (trial registry number: NCT04618289 – www.clinicaltrials.gov). All information collected for the study will be kept strictly confidential, anonymized and all participants will be allocated a numerical code. Only the researchers conducting the study will have access to such information, and the data will not be shared with any party without the participants’ consent. Furthermore, the data will be collected at UPM, saved in the university network and will be password protected. Moreover, the blood samples will also be anonymized and stored in a freezer located in the laboratory in the Department of Nutrition, UPM. The findings will be written up for publication in peer-reviewed journals and presented at national or international conferences. No individual from the study will be identified in any subsequent report, presentation, or publication. In addition, participants will be awarded a cash honorarium upon completing the study but will not be reimbursed with any other expenses (including travelling) incurred while participating in the study.

## Discussion

Vitamin D has been shown to play a role in calcium [37] and phosphate homeostasis [38], bone health[39] and other human physiological systems, including cardiovascular, brain, pancreas, muscle and immune system, despite the inconsistent findings [39]. Besides, its potential role has recently been suggested in erythropoiesis, especially in linking hemoglobin and serum 25(OH) concentrations in humans, but with limited evidence [17]. Furthermore, the underlying mechanism behind vitamin D’s effect on iron regulation is poorly understood [40]. Besides, the proposed theory was based on observations reported in studies carried out in patients with disease complication-derived anemia, such as hemodialysis patients [41, 42] or critically ill patients [43]. The proposed theory revolves around vitamin D’s mechanism that affects hepcidin expression, pro-inflammatory cytokine production, and erythropoiesis rate. Erythroid precursor cells are responsible for expressing 1,25(OH)D receptors, which stimulates progenitor cell production and development required in erythropoiesis. Consequently, a compromised concentration of the vitamin D active metabolite, 1,25(OH)D, will then change the rate of erythropoiesis [17, 44]. This active metabolite has also been suggested to improve the responsiveness of patients administered with erythropoiesis-stimulating agent (ESA) in managing anemia by improving erythropoiesis, thus decreasing the use of ESA when supplemented with vitamin D [45]. In addition, the involvement of inflammatory cytokines in the proposed mechanism is particularly related to anemia due to inflammation, mostly in chronic disease patients. The release of inflammatory cytokines leads to increased hepcidin concentration, known for its role as an iron regulator [19]. The use of vitamin D has been shown to decrease hepcidin expression and increase ferroportin to export more iron and ultimately decrease the incidence of anemia [40].

Iron deficiency based on plasma ferritin concentration of < 15 μg/l in women aged 18–40 years in Malaysia was reported to be approximately 33%, and anemia is currently 29.9% in the reproductive-aged women group in the recent national survey. Despite the reduction in anemia incidence as opposed to 34.5% in 2015, anemia remains a public health issue not only in Malaysia but worldwide; therefore, findings from RCTs will be clinically relevant. Despite numerous approaches implemented, including dietary modification, iron fortification and supplementation, iron deficiency and anemia are still prevalent in at-risk groups. Each strategy comes with its advantages and disadvantages. Vitamin D utilization has been recently highlighted as a novel iron absorption enhancer that acts through suppressing hepcidin, the main iron regulator, albeit the paucity of intervention studies, especially RCTs. Previous research was mostly (1) cross-sectional studies that cannot establish a causal-effect relationship, utilizing bolus concentrations of vitamin D or fortified in a food vehicle, (2) carried out in patients, and cannot be generalized to the healthy population, or (3) in vitro studies which cannot be directly extrapolated to humans. To the best of our knowledge, this study will be the first RCT to investigate the utilization of vitamin D-fortified juice, consumed daily as part of the diet to recover the iron status in a healthy pool of Malaysian women with marginally low iron stores. The clinical aspects findings in the present study may be relevant to the recovery of iron status in the iron-deficient population by using vitamin D supplementation in food fortification as a novel iron absorption enhancer in the future. The study also will measure hepcidin concentration which may provide information on the mechanism of vitamin D action on iron recovery status, which was not measured in previous intervention studies. The study is limited by not including a specific measure of adherence for the consumption of fruit juices; however, participants will be required to return the used sachets and food diaries during the interim and post-intervention time points to account for compliance. The study will not measure the circulating 25(OH)D concentrations in blood samples using liquid chromatography-tandem mass spectrometry, which is the reference method to quantify vitamin D active metabolite.

## Supporting information

S1 Checklist SPIRIT checklist. (DOC)

S1 File Approved clinical study protocol. (PDF)
