# Supplementary material for: Effect of vitamin D3-fortified fruit juice supplementation of 4000 IU daily on the recovery of iron status in childbearing-aged women with marginally low iron stores: Protocol for an 8-week, parallel group, double-blind randomized controlled trial
Source: PLoS One. 2022 Mar 25;17(3):e0265772. doi: 10.1371/journal.pone.0265772 (PMC8956161; doi:10.1371/journal.pone.0265772)
Supplement: S1 File — (PDF) [file pone.0265772.s002.pdf]

PROPOSAL

Document submitted for ethical approval from JKEUPM 2020

1. Research Title :	Effect of vitamin D3-fortified fruit juice supplementation on the recovery of iron status in childbearing-aged women with marginally low iron stores
2. Principal Investigator :	Dr. Salma Faeza Binti Ahmad Fuzi
3. Co- Investigators :	1. Assoc. Prof. Dr Loh Su Peng<br>2. Madam Norhafizah Ab Manan<br>3. Professor Dr Muhammad Najib bin Mohamad Alwi

4. Introduction :

Research background including background, literature review, problem statement and rational of study

Anaemia is one of the most common micronutrient deficiencies, affecting populations worldwide (WHO, 2008) and 50% of anaemia occurrences were reported to be caused by iron deficiency, leading to multiple negative aspects to human health. A total of approximately 800 million women and children are anaemic in 2011, represented nearly 530 million women and 270 million children, and 50% of them were reported to also be iron deficient (WHO, 2015). It was reported that the overall national prevalence of anaemia in Malaysia was 24.6%, which was found to be higher in women (35.5%) as opposed to men (14.3%) (NHMS, 2015). Anaemia prevalence at the national level for general population is lacking, especially for women of childbearing aged, however, an earlier multi-centre study carried out by Haniff et al. (2007) showed that the prevalence of anaemia (haemoglobin concentration < 11 g/dl) was 35% in 1072 pregnant women aged 16-54 years recruited in 56 antenatalclinics in Malaysia. Combatting anaemia or iron deficiency requires cohesive approach, as its occurrence is suggested to be multifactorial. Iron supplements have been widely used in at-risk groups, whereas, either dietary pattern modification or iron fortification may be implemented at population levels (WHO, 2008). Extensive literature, including scientific evidence from systematic reviews is available on the effect of dietary-based approach and iron supplementation on the improvement of iron status, reporting inconsistent findings (Table 4.1).

Table 4.1. Recent experimental studies investigating the effect of different types of interventions on the recovery of iron status

Study	Participant	Intervention	Main findings
A) Dietary modification			
Blanton et al., 2013<br><br>United States	Women, n= 43<br><br>18-30 years	Beef or non-beef lunch<br><br>16-week	No significant difference between intervention and control groups in alliron status indices.
McArthur et al., 2012	Women, n=76	A: Pork-diet + placebo	SF concentration significantly higher at post-intervention in C compared to A &

Australia	18-35 years	B: Control diet + placebo<br><br>C:Control diet + supplement<br><br>12-week	B groups.
B) Iron fortification			
Karl et al., 2010<br><br>United States	Women, n=142<br><br>21 ± 4 years	Iron-fortified food bars or placebo food bars<br><br>9-week	Hb concentration significantly higher at post-intervention in the intervention group compared to placebo.
Andersson et al., 2010<br><br>Switzerland	Women, n=142,<br><br>18-40 years	A: Fortified margarine (FP)<br><br>B: Fortified margarine (Na)<br><br>C: Placebo margarine<br><br>32-week	SF concentration significantly higher at post-intervention in B compared to Aand C groups.
Blanco-Rojo et al., 2011<br><br>Spain	Women, n=130,<br><br>18-35 years	Iron-fortified juice or placebo juice<br><br>16-week	SF concentration significantly higher at post-intervention in the intervention group compared to placebo.
Beck et al., 2011<br><br>Australia	Women, n=89,<br><br>18-44 years	A: Iron-fortified cereal + kiwi<br><br>B: Iron-fortified cereal + banana<br><br>16-week	SF concentration significantly higher at post-intervention in A compared to B.
C) Iron supplementation			
Hoppe et al., 2013<br><br>Switzerland	Women, n= 77<br><br>¥24 years	A: haem blood-based bread<br><br>B: ferrous fumarate 35mg<br><br>C: ferrous fumarate 60mg<br><br>D: iron-free supplement<br><br>12-week	No significant difference between A and B, C, D groups in all iron status indices.
Ortiz et al., 2011<br><br>Colombia/Argentina	Pregnant women, n=80<br><br>¥>16 years	A: iron poly maltose (IP)<br><br>B: ferrous sulphate (FS)<br><br>12-week / 100 mg	SF concentration significantly higher at post-intervention in group A compared to group B.
Vaucher et al., 2012<br><br>France	Women, n=198<br><br>18-50 years	A: ferrous sulphate (FS)<br><br>B: placebo	SF and haemoglobin concentrations significantly higher at post-intervention in group A compared to group B.

		12-week / 80 mg	
Berber et al., 2014<br>Turkey	Women, n=104<br>19-60 years	A: iron succinylate flacon<br>B: iron glycine sulphate<br>12-week / 80 mg	Haemoglobin concentration significantly higher at post-intervention in iron ferrous group (B) compared to iron ferric group (A)

SF: Serum ferritin; Hb: haemoglobin; FP: ferric pyrophosphate; Na: sodium iron edetate or NaFeEDTA; ¥SD and range for age not available

In a systematic review by Casgrain, Collings, Harvey, Hooper, and Fairweather-Tait (2012), it was found that added iron from supplements, fortifiers or dietary source improved iron status, reflected by increased in haemoglobin (0.51 g/dl, CI: 0.37, 0.65 g/dl, p<0.00001) and serum ferritin concentrations (9.19 µg/l, CI: 6.63, 11.75 µg/l, p<0.00001). Gera, Sachdev, and Boy (2012) carried out another meta-analysis replicated the findings by demonstrating the effectiveness of iron fortification intervention in improving haemoglobin (0.42 g/dl, CI: 0.28, 0.56, p<0.001) and serum ferritin (1.36 µg/l, CI: 1.23, 1.52 µg/l, p<0.001) concentrations at post-intervention. It is well established that wide range of studies have been carried out addressing different strategies in managing anaemia, ID or IDA. In Malaysia, however, there is scarcity in published evidence,especially on the iron status of non-pregnant childbearing-aged women.

Altering individual's preference of food has been one of the barriers to improving iron status through dietary modification (Lynch, 2011), and iron fortification did not completely improved general iron status (Pasricha, Drakesmith, Black, Hipgrave, &Biggs, 2013). The efficacy of oral iron supplementation is limited due to its adverse effects causing low adherence which leads to inefficiency of the intervention (Souza, Batista Filho, Bresani, Ferreira, & Figueiroa, 2009) and its low bioavailability (Ferreira da Silva, Dutra-de-Oliveira, & Marchini, 2004).

A clearer understanding of the interaction between inflammation, erythropoiesis, and hypoxia, which is regulated by the iron regulator peptide hormone, hepcidin may benefit in designing effective iron interventions that result in fewer adverse effect with utmost advantage (Pasricha et al., 2013). In reference to this, vitamin D has recently been suggested to play role in erythropoiesis process, but there is limited evidence available, especially in human studies (Sim et al., 2010). Vitamin D has been demonstrated in previous studies to be capable of increasing proliferation of erythroid precursors in the bone marrow to support erythropoiesis by decreasing the expression of pro-inflammatory cytokines which cause the suppression of hepcidin. Decreased cytokines and suppressed hepcidin leads to higher iron bioavailability for RBC production and haemoglobin synthesis (Smith et al., 2016). A recent review by Smith and Tangpricha (2015) also found that the use of vitamin D may facilitates in the anaemia occurrence via erythropoiesis. Vitamin D, which is a secosteroid hormone, existed in two major forms; D3 or cholecalciferol and D2 or ergocalciferol (SACN, 2016), which both utilised in vitamin D-fortified foods and supplements (Holick et al., 2011). Anaemia and vitamin D deficiency are two of the most widespread nutritional problems, however, the mechanism that linked these deficiencies remains unclear, and there is a scarcity of evidence that supports the links between these deficiencies in the normal population, as previous studies were predominantly carried out in hospital

patients (Han et al., 2013). Recent observational studies carried out in various settings and populations have suggested an inverse association between increased anaemia risk and serum/plasma 25(OH)D concentrations (Ernst et al., 2016). Inconsistent findings on the link between vitamin D deficiency and iron deficiency ; for instance, vitamin D deficiency was shown to results in higher risk of anaemia in participants aged >17 years (Sim et al., 2010) but iron deficiency and anaemia were not found to be associated with vitamin D deficiency in children (McGillivray et al., 2007) have been reported. The mechanism that links these deficiencies remains unclear, as there are inconsistent and limited number of published articles that show administration of vitamin D may or may not affect the stimulation of erythroid precursors and ultimately rate of erythropoiesis (Yoon, Kim, Yoo, & Kim, 2012). There is a scarcity of evidence that supports these findings in the normal population as previous studies were predominantly carried out in kidney disease, heart failure or diabetic patients (Han et al., 2013). Vitamin D deficiency has been shown to be associated with not only IDA, but to different types of anaemia, as complications due to disease, such as anaemia of chronic kidney disease (ACKD) in kidney disease patients or anaemia due to infections in chronic illness patients such as cancer or inflammatory disorders (Sharma, Jain, & Dabla, 2015).

Association between vitamin D deficiency and a higher risk of anaemia was reported in limited number of cross-sectional studies carried out in multiple settings. For instance, anaemia prevalence in the vitamin D deficient group (48.8%) was found to be significantly higher in the non-deficient group (35.7%, $p<0.01$) in a study carried out by Sim et al., (2010) in 11 medical centres in California, USA among adolescents and adults of both sexes aged 17+ years. A weak but significant linear association between haemoglobin and serum 25(OH)D concentrations ($r=0.221$, $p=0.026$) was observed in another cross- sectional study carried out in 102 Korean children aged 3-24 months who attended a medical centre in Korea (Jin, Lee, & Kim, 2013). In this study, it was observed that vitamin D deficiency was more predominant in the IDA group (67%) compared to ID (53%) and normal (29%) groups. In a larger scale of cross-sectional study, Lee et al. (2015) reported that the incidence of vitamin D deficiency was higher in anaemic female participants (65%), compared to only 40% anaemic male participants, which lead to an to increased risk of anaemia. A cross-sectional study which included data obtained from 263 children aged 3 months-12 years who attended an outpatient department of a hospital in New Delhi, India reported a higher anaemia incidence in vitamin D deficient participants (66%) compared to vitamin D sufficient participants (35%) (Sharma et al., 2015). A moderate, but significant linear association ($r=0.317$, $p=0.013$) between serum 25(OH)D and haemoglobin concentrations was observed. A multiple regression test carried out discovered that there was an increased risk of vitamin D deficiency in participants who were anaemic ($p<0.001$) (Sharma et al., 2015), also consistent with findings from the previous studies. However, it should be noted that different observational studies have employed various thresholds, especially in defining vitamin D status as there is no established international threshold. The different thresholds used in each study may have an effect on the reported figures of prevalence, causing difficulty in the comparison and interpretation.

To the best of knowledge, there were only two studies to date that combined in-vitro and in-vivo studies investigating the potential mechanism of how vitamin D plays role in affecting iron homoeostasis by regulating transcription of HAMP gene that responsible in hepcidin production which is known to its function as an iron regulator. It was observed in an in-vitro study by (Bacchetta et al., 2013) that hepcidin expression was significantly reduced by at least 0.4-fold when treated with 25(OH)D and 1,25(OH)D ($p<0.001$) compared to control vehicle treatment, and it was suggested that it was a direct inhibition of 25(OH)D and 1,25(OH)D on transcription of the HAMP gene. Based on these findings, Bacchetta et al. (2013) carried out a pilot humanstudy using 7 healthy participants administered orally with vitamin D2 (100 000 IU). Evidence from this human study supported the in-vitro findings, that serum hepcidin concentration significantly reduced to 34% following 24 hours of supplementation ($p<0.05$). Analysis of serum for the next 72 hours of supplementation showed that the serum hepcidin concentration was further decreased to 33% ($p<0.01$). Small sample size is the limitation of this pilot study, but the significant decline observed in serum hepcidin concentration despite the small sample size, in addition to the evidence from the cell study was sufficient to initiate bigger scale investigation especially RCTs.

In another study by Zughaier, Alvarez, Sloan, Konrad, and Tangpricha (2014), hepcidin expression was found to be significantly suppressed to below 10-fold (LPS/20 nM 1,25-OHD) and below 5-fold (LPS/40 nM 1,25-OHD) compared to control, both with $p<0.05$. For the pilot controlled trial in human, Zughaier et al. (2014) included 38 kidney patients with stage 2 and 3 ACKD, who were randomised to receive oral administration of vitamin D3 (50 000 IU/week), and every other week for 40 weeks or placebo for 12 weeks. Moderate but significant negative association was observed between the percentage of changes in serum hepcidin and serum 25(OH)D concentrations ($r=-0.38$, $p=0.02$) ($n=38$). The study demonstrated that bolus administration of vitamin D3 affected the changes in serum hepcidin concentration, but the study was carried out in kidney patients commonly associated with anaemia, and results cannot be generalised to the general population, healthy or iron deficient.

Despite numerous approach implemented, iron deficiency are still prevalent, especially in the at-risk group. Each strategy comes with its advantages and disadvantages; and there is recently emerging evidence of the utilisation of vitamin D, as iron absorption enhancer that acts on suppression of hepcidin, the main iron regulator. However, there is a scarcity of randomised controlled trial, investigating the effect of vitamin D supplementation using fruit juice as the food fortification vehicle, administered routinely, aiding as an iron absorption enhancer, on iron status especially in the general population who are at risk of iron deficiency, not only in Malaysia but worldwide. Previous studies were mostly cross-sectional studies which cannot establish a causal-effect relationship; utilising bolus concentration of vitamin D or fortified in food vehicle; carried out in patients and cannot be generalised to the general population, or cell studies which cannot be directly extrapolated to human systems.

5. Research questions :

Therefore, the present study is designed to utilise vitamin D3-fortified fruit juice as supplements that may potentially act as an iron absorption enhancer to improve iron status in the Malaysian child-bearing aged women with low iron stores. In addition to investigating the efficacy, this study is also designed to assess the effect of a higher dose of vitamin D3 in the fortified fruit juice (4000 IU) on iron metabolism. The study will include the measurement of plasma hepcidin and 25(OH)D concentrations to investigate a possible mechanism that links vitamin D and iron deficiency, as postulated from the existing literature. For that reason, the aim of the study is to investigate the effect of an 8-week vitamin D3-fortified fruit juice supplementation on iron status indicators, including hepcidin concentration in childbearing aged Malaysian women with marginal iron stores. It is hypothesised that there will be a significant improvement in haematological indicators following 8-week daily vitamin D3- fortified fruit juice supplementation in the vitamin D group compared to placebo group. It is also hypothesised that plasma hepcidin concentration will be reduced following 8-week daily vitamin D3-fortified fruit juice supplementation, which results in increased iron stores.

6. Research objectives and hypotheses :

General objective

To investigate the effect of an 8-week vitamin D3-fortified fruit juice supplementation (4000 IU) consumed daily on haematological indicators in a cohort of marginally low iron stores childbearing-aged Malaysian women.

Specific objectives

- To examine the effect of vitamin D3-fortified fruit juice supplementation, consumed daily on the recovery of iron status in women with marginal low iron stores
- To investigate the effect of vitamin D3-fortified fruit juice supplementation on plasma hepcidin concentration, to have better understanding of the potential role of vitamin D as an iron absorption enhancer
- To investigate the effect of vitamin D3-fortified fruit juice supplementation on plasma 25(OH)D concentration, to have better understanding of the potential role of vitamin D as an iron absorption enhancer
- To examine the body weight status and dietary intake of women with marginal low iron stores

7. Literature Review : Addressed in Section 4 (Introduction)

8. Conceptual Framework :

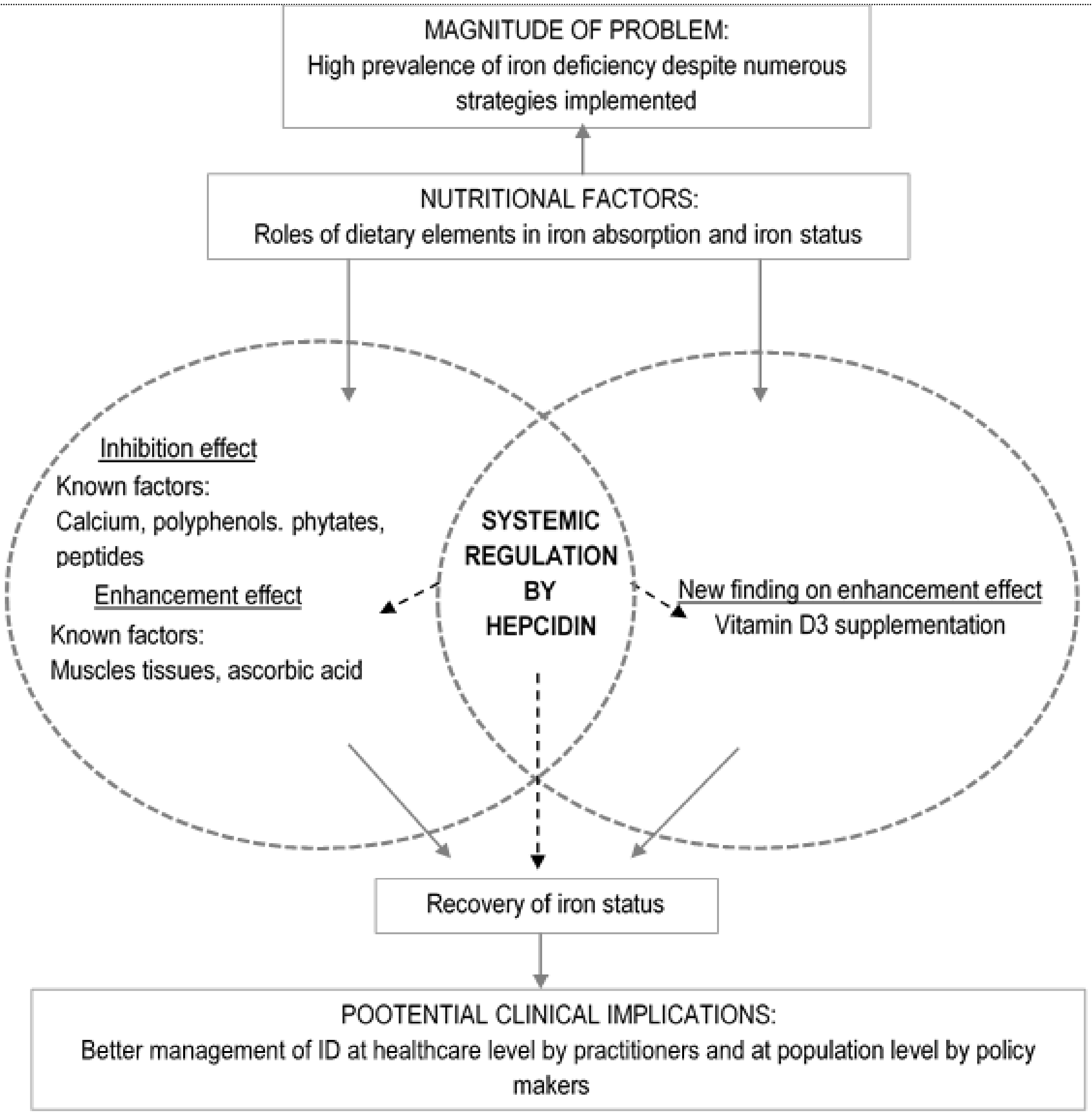

9. Research Methodology :

9.1 Participants

9.1.1 Exclusion and inclusion criteria

The inclusion criteria will be women of child bearing age, healthy, aged 19-40 years, and non-pregnant nor lactating. The women will be excluded if they had a history of gastrointestinal disorder (celiac disease, Crohn's disease, irritable bowel syndrome gastroesophageal reflux disease, peptic ulcers and other related gastrointestinal disorders which may cause nutrient malabsorption) and iron metabolic disorders such as iron overload, had donated blood since the past 6 months, and regularly consuming nutritional supplements (iron, vitamin D, vitamin C, calcium).

9.1.2 Sample size

Sample size was estimated by using (mean ± S.D) serum ferritin concentrations (ug/dl) from a randomised trial carried out by Blanco Rojo et al. (2011) in 41 women aged 18-35 years with low iron stores to determine the effect of iron-fortified fruit juice consumption on both iron and vitamin D status. At week 8 of study, it was observed that (mean ± S.D) serum ferritin concentrations were significantly higher in the vitamin D group (34.1 ± 14.8 ug/l), compared to 24.8 ± 17.7 ug/l in the placebo group ($p<0.05$). Based on this study, the effect size is calculated by dividing the mean difference (9.3 ng/ml) by the pooled standard deviation (16.29). With an effect size of 0.571, the total sample size will be required in the present study is 50/group (Power=0.80, α err prob=0.05). Allowing for a 20% drop-out rate, the total sample size required to demonstrate a significant difference in serum ferritin concentrations between vitamin D group and placebo is estimated to be 120 (60 participants/group). The sample size was estimated using of G-Power Software (Version 3.1.7).

9.1.3 Recruitment and screening

The prevalence of iron deficiency based on plasma ferritin concentration of < 15 µg/l in women aged 18-40 years in Malaysia was reported to be approximately 33% (Loh & Khor, 2010). The prevalence of adult women who consumed vitamin and mineral supplements (VMS) based on the Malaysian Nutrition Adult Survey (MANS) 2014 was reported to be approximately 32.1% (Mohd Zaki et al., 2018). To take into account the combination of both data, approximately 736 women would have to be screened to achieve the required sample size. Participants that will be recruited includes childbearing-aged women who are working and staying in Serdang, Kajang and Bangi, Selangor areas. The potential participants will be recruited from either schools, universities, residential areas or government officials in Serdang, Kajang and Bangi areas. Medium such as posters, flyers, emails will be sent to various departments within universities or government officials, discussion boards near residential areas, social media pages and university/government officials' webpage/intranet.

The potential participants will be then screened to determine their plasma ferritin and haemoglobin concentrations to include women with marginal low iron stores [plasma ferritin concentration < 20 µg/l] (Ahmed, Coyne, Dobson, & McClintock, 2008; McDonnell & Witte, 1997) and non-anaemic [haemoglobin concentration > 12 g/dl] (WHO/UNICEF/UNU, 2001).

9.2 Study Design

The study is a placebo controlled, double-blind randomised controlled trial, designed to investigate the effect of an 8-week vitamin D3-fortified fruit juice supplementation on haematological indicators and hepcidin response in a cohort of marginally-low iron stores Malaysia childbearing-aged women. The study will be registered with the NMMR and at clinicaltrials.gov and ethical approval for the study protocol will be obtained from the Ethic Committee for Research Involving Human Subject UPM (JKEUPM) (Figure 9.1) (Appendix I). The study will be carried out for the duration of 8 weeks in Selangor, Malaysia. The data will be collected between June 2020 and June 2021. Figure 9.2 outlines the study design which included 2 main phases of the study (Appendix II). Phase 1 will be the recruitment and screening phase when potential participants will be screened for marginally low iron stores, identified and randomised. Phase 2 will be the intervention phase where all the eligible participants

will be required to consume either vitamin D3-fortified fruit juice containing 4000 IU (100 mcg) (vitamin D group) or placebo-fruit juice (placebo group) daily for the duration of 8 weeks.

9.2.1 Phase 1: Screening

All participants will be provided with a participant information sheet (PIS) and a date will be arranged for the venous blood collection to ascertain the plasma ferritin and haemoglobin concentrations, which will be used to indicate iron stores and haemoglobin levels in the present study. The potential participants will be given a written informed consent and will be asked to complete a screening questionnaire to ensure eligibility criteria is met. The screening questionnaire will be used to obtain socio-demographic characteristics including age, ethnicity, religion, educational level and household income. During the screening clinic session, 1 ml of venous blood will be collected at the clinical laboratory within the Department of Nutrition & Dietetics at the UPM by the researcher who is a trained phlebotomist. The whole blood and plasma sample collected will be used to determine the concentrations of ferritin and haemoglobin. Based on the blood analysis, participants will be notified of their results via email or phone. The eligible participant who has marginally low iron stores (based on plasma ferritin concentration of between < 20 ug/l) and non-anaemic (based on haemoglobin concentration of > 12 g/dl or higher) will be then invited to continue to Phase 2 of the study. Participants who had abnormally high plasma ferritin concentrations (> 150 ug/l) and low haemoglobin concentrations (< 12 g/dl) from the screening will be advised to arrange a check-up with their registered general practitioner and not included in the study. An initial testing of fruit juices will be carried out during the baseline clinic session for participants to test the palatability of the fruit juices.

9.2.2 Phase 2: Intervention

A total of 120 participants who are eligible to continue with the study will be randomised to receive either 4000 IU (100 mcg) of vitamin D3-fortified fruit juice or placebo-fruit juice. The participants will be instructed to consume the vitamin D3-fortified fruit juice or placebo-fruit juice in the morning by diluting the powder in 150 ml of water, daily, for the 8-week duration of the study. All the participants will be reminded to not alter their dietary habits and physical activity, in addition, to abstaining from donating blood during the course of the study which may interfere with interpretation of study findings.

9.2.3 Study clinic sessions

Participants will all be required to attend clinic sessions after overnight fasts of approximately 8 hours and will be expected to attend 3 clinics in total for the duration of study. The clinic sessions will be in the clinical laboratory within the Department of Nutrition & Dietetics at the UPM where the initial screening session carried out. Participants will be asked to consume only water during the overnight fast. Each clinic will last approximately 30 minutes and will take place between 8-10 am in the morning.

The details of each clinic sessions are as follows.

Clinic 1 (Week 0, baseline): Height (cm) will be measured by using digital stadiometer and weight (kg) using weighing scales, followed by collection of a 10 ml fasted venous blood sample collection. Participants will be provided with a 3-day food diary to be completed at home before the clinic and collected during the clinic. An 8-week supply of a supplement pot, containing fruit juice powder in sachets with assigned participant ID will be provided to the participants. The protocol will be explained to participants and an email will be sent as a reminder to the participants one day before the following clinic.

Clinic 2 (Week 4, interim): The same procedure will be repeated except for measurement of height. Participants will be then be given a further 3-day food diary to be completed before the next clinic.

Clinic 3 (Week 8, post-intervention): The same procedure will be repeated except for measurement of height. The final 3-day food diary and supplement pot (with any) of the remaining fruit juice powder in sachets to calculate the compliance will be collected.

9.3 Randomisation and blinding of participants

4.3.1 Randomisation

The randomisation process will be carried out using a computer-generated software (www.randomization.com) by a third party, independent to the study. Participants (n=120) will be randomised to 2 groups: vitamin D3-fortified fruit juice (vitamin D group) or placebo-fruit juice (placebo group).

The third party will allocate 62 D3-fortified fruit juice sachets and placebo-fruit juice sachets into each supplement pot according to the generated plan. An excess of 6 sachets in each pot will enable the researcher to estimate compliance once the study is completed. Each supplement pot will then be sealed in a tamper proof supplement pot and numbered ready to be provided to participants.

4.3.2 Blinding

The participants and researcher will be double-blinded as to which groups participants will be assigned. The researcher will administer the numbered supplement pot (i.e; 001) to the participants based on the sequence that participants attend their baseline clinic (Week 0). The blinding will be maintained throughout the study period of 8 weeks and allocation will not be unlocked until the end of the data analysis or during any adverse event.

9.4 Vitamin D3-fortified fruit juice supplement and placebo

The vitamin D3-fortified fruit juice supplement that will be used is vitamin D3 cholecalciferol (4000 IU, 100 mcg, Fiatec Biosystem Sdn Bhd, Selangor, Malaysia). The matching placebo will be also custom-produced and will be produced in the same manner, without the active ingredients by the same company. The placebo produced will match with vitamin D3 in terms of appearance, size, colour and taste to achieve the double-blind design.

9.4.1 Assessment of compliance

Compliance to the supplementation (%) will be calculated based on the following formula: (62 – remaining sachets in the pot)/56 x100. The participants will also be required to retain and hand over the sachets used during the 3 clinic sessions to also assess the compliance. For the total duration of 8 weeks, participants will be required to consume 56 capsules of vitamin D3-fortified fruit juice or placebo-fruit juice.

9.4.2 Adverse effect and safety

The vitamin D3-fortified fruit juice powder supplements will be packaged in individual sachet for daily consumption and produced specific to research requirement and in accordance to Good Manufacturing Practice (GMP) standards. For safety purposes, powder produced will be sent for microbiological tests and heavy metals for it to be deemed safe for human consumption. These tests will be carried out on the powder before it is packaged into sachets and in the finished products.

The vitamin D recommended intake in different populations worldwide is currently set to between 100-800 IU (Adams & Hewison, 2010), and there is no international consensus on the optimal dietary intake of vitamin D (Brouwer-Brolsma et al., 2013). The range between 100-800 IU is regarded as underestimated, contributes to the difficulty in maintaining sufficient vitamin D status, hence, the need for additional vitamin D from supplement form (Adams & Hewison, 2010). The RNI proposed includes the daily intake from either naturally-contained vitamin D food sources, vitamin D-fortified foods or supplements. Table 9.1 summarises the recommended dietary and supplement intakes from various sources worldwide.

Table 9.1 Overview of vitamin D dietary and supplement recommendations for adults

Reference	Dietary	Supplements (D2 or D3)
UK SACN (SACN, 2016)	400 IU/day (10 µg)	400 IU/day (10 µg)
IOM (Ross et al., 2011)	600 IU/day (15 µg)	600 IU/day (15 µg)
The Endocrine Society (Holick et al., 2011)	600 IU/day (15 µg)	6000 IU/day (150 µg) or 50000 IU/week (1250 µg) (8 weeks)
Vitamin D Council	5000 IU/day (125 µg)	5000 IU/day (125 µg)
Vieth (2005)	200 IU/day (5 µg)	4000 IU/day (100 µg)

The dose of 4000 used in the study is a safe dose for adults and not associated with any adverse effect. Various institutions recommended different safe dosages between 400-6000 IU/day or up to 50000 IU/week (Holick et al., 2011; Ross, Taylor, Yaktine, & Del Valle, 2011; Vieth, 2005). Published studies investigating the use of vitamin D supplements or fortified food products on iron and vitamin D status biomarkers using various doses have not reported adverse events (Appendix III).

9.5 Anthropometric measurements

9.5.1 Height

Height will be measured to the nearest 0.1 cm with a wall mounted digital stadiometer (Model 264 SECA, Hamburg, Germany). Before the start of measurement, participants will be checked to ensure minimal clothing and barefoot to allow accurate positioning. With the head positioned at Frankfurt horizontal plane, participants will be instructed to stand with the legs straight, heels closed together, shoulders relaxed, and arms at the side. The 4 points, which included back of the head, buttocks, heels and scapulae, will be checked to ensure they are in line with the vertical surface of the stadiometer. The reading will be recorded when the headboard is lowered to the highest point of participants' head. The measurement will be performed in duplicate and instrument used will be calibrated to ensure accuracy (Lee & Nieman, 2010).

9.5.2 Body weight

Body weight will be measured to the nearest 0.1 kg using an electronic scale (Model 875 SECA, Hamburg, Germany). The scale will be set on a flat and hard surface to ensure stability when the measurement is made. Before the measurement, participants will be asked to be barefoot, empty out the pockets and wear minimal clothing. To ensure even distribution of body weight on both feet, participants will be instructed to stand still in the middle of the platform scale without touching anything. The measurement will be performed in duplicate and instrument used will be calibrated to ensure accuracy (Lee & Nieman, 2010).

9.5.3 Body mass index (BMI)

The BMI will be calculated using height and weight obtained from the anthropometric measurement of the participants. The cut off values to define BMI are summarised in Table 9.2

Table 9.2 Classification of BMI for Asians (WHO, 2004)

Classification of BMI	BMI (kg/m^2)
Underweight	< 18.5
Normal	18.5 – 23.0
Overweight	23.0 – 27.5
Obesity	≥27.5

9.6 Dietary Intake Assessment

Participants will be required to complete a 3-day food diary at baseline and post intervention (to include 2 weekdays and 1 weekend day) to estimate their habitual dietary intake. The diary is comprised of instructions on how to appropriately record participants' dietary intakes, including a guide to portion sizes, how to describe the foods/drinks in detail, together with a sample diary. In each section of the diary, there will be columns for time of consumption, location, description of food/drink consumed, brand and amount/quantity, physical activity and recipe sections for the participants to complete. Dietary records will be analysed for nutritional content by using Nutritionist Pro Software (Axxya System LLC, Washington, USA). The food items used for the analysis will be derived from Malaysian Food Composition Table (Tee, Ismail, Nasir, & Katijah, 1997) and USDA. The Malaysian Recommended Nutrient Intake (RNI 2017) will be used as a reference to compare the intake of energy,macro, and micronutrients of the participants. Table 9.3 shows the RNI values that will be used in the study to compare to the dietary intake of participants.

Table 9.3 RNI/day for females aged 19-50 years

Nutrient/day	Age groups	Malaysia RNI (2017)
Energy (for PAL of 1.6)	18-29 years	1840 kcal
	30-59 years	1900 kcal
Protein	18-29 years	53 gram/day
	30-59 years	52 gram/day
Total carbohydrate		50-65% total energy intake
Total fat	19-29 years	51-61 gram/day
Iron	19-50 years	29 mg/day (10%), 20 mg/day (15%)
Vitamin D	19-50 years	15 μg

9.7 Sun exposure quantification

A set of validated and modified questionnaire will be used (Detert et al., 2015; Hansen et al., 2016) to account for sun exposure which may confound study findings. The questionnaire includes section on sun habits and protection, which is scored based on a 5-Likert scale. The range of score is between 0-32 which indicates higher risk exposure with a higher score (Detert et al., 2015). Participants will also be required to record the hours they spend under the sun during the day for the duration of study.

9.8 Venepuncture Procedure

9.8.1 Venepuncture protocol and blood handling

Venepuncture will be performed by a trained phlebotomist (with observation by a medical doctor who is part of research team) to obtain 10 ml venous blood samples from participants at each clinic. Both whole blood and plasma obtained from the venepuncture will be used in the study for analysis. The blood sample will be collected in lithium heparin and EDTA blood collection tubes (BD Company, New Jersey, USA) depending on the blood biomarker analysis. Whole blood will be used immediately after each clinic to measure full blood counts (FBC). Venous blood samples collected will be centrifuged for 10 minutes (1600 g) at 4°C to obtain plasma samples required for iron and vitamin D biomarkers which will then be aliquot into microcentrifuge tubes and stored at -80 ^{o}C before being used for analysis. Plasma samples will be used to analyse iron status biomarkers (C-reactive protein, ferritin and hepcidin) and vitamin D metabolism biomarkers (25-OH D) concentrations.

9.9 Analysis of Blood Samples

4.9.1 Determination of full blood counts (FBC)

Measurement of FBC will be carried out using an automated Beckman Coulter Ac.T diff Haematology Analyser (Bechman Coulter Inc California, USA. The normal values/ranges that will be used in the study are summarised in the Table 9.4.

Table 9.4 Normal range of full blood count concentration for adult women (Kaushansky et al., 2015)

Full blood counts (FBC) indicators	Unit	Normal values
RBC count	$\times 10^{12}$/L	3.8 – 5.2
Haemoglobin concentration	g/dL	11.5 – 15.5
Haematocrit/packed cell volume (PCV)	L/L	0.37 – 0.47
Mean corpuscular volume (MCV)	fl	80 - 96
Mean corpuscular haemoglobin (MCH)	pg	27 – 32
Mean corpuscular haemoglobin concentration (MCHC)	g/dL	32 – 36
White blood count (WBC)	$\times 10^{9}$/L	4 - 11

9.9.2 Determination of iron and vitamin D status biomarkers

Commercially available ELISA assay kits will be used in the determination of iron and vitamin D status biomarkers. This includes the measurement of plasma C-reactive protein (CRP) (R&D Systems Inc, Minneapolis, USA), ferritin (Elabscience Biotechnology Co. Ltd), and hepcidin (R&D Systems Inc, Minneapolis, USA) concentrations for iron status biomarkers. Biomarkers including 25(OH)D (Calbiotech Inc, California, USA) concentration will be determined for vitamin D status.

Table 9.5 shows the normal thresholds that will be used to define normal ranges of the specified blood biomarkers.

Table 9.5 Normal thresholds of iron and vitamin D status biomarkers

Biomarkers	Normal thresholds	Reference
Ferritin	> 15 µg/l	WHO/UNICEF/UNU (2001)
CRP	<3-10 mg/l	WHO/CDC (2004)
Hepcidin	17-286 ng/ml	Ganz, Olbina, Girelli, Nemeth, and Westerman (2008)
25(OH)D	>50 nmol/l	Ross et al. (2011)

10. Data Analysis :

All statistical analyses will be performed using IBM SPSS Statistic Data Editor Software (Version 21). Shapiro-Wilks or Kolmogorov-Smirnov tests will be used to determine the normal distribution of data as appropriate. Descriptive statistics will be used to describe frequencies, means and standard deviations. Baseline characteristics comparisons between the groups (vitamin D and placebo groups) will be carried out using independent t-test for normally distributed or Mann-Whitney test for non-normally distributed data. Mixed model repeated measures analysis of variance (ANOVA) will be performed to determine the effect of intervention and the interaction with time points for all iron status and vitamin D status blood biomarkers. The changes in all iron status and vitamin D status blood biomarkers from baseline to post-intervention (week 8) between the two groups will be compared and analysed using independent t-test for normally distributed or Mann-Whitney test for non- normally distributed data. Analyses will be carried out for all participants who are randomised, irrespective of compliance to study protocol (intention-to-treat). Differences will be considered significant with a p-value ≤ 0.05.

11. Expected outcome :

- Prevalence of low iron stores and vitamin D insufficiency in premenopausal childbearing-aged women in Selangor
- Potential role of vitamin D supplements as one of the new iron absorption enhancers in the recovery of low iron stores
- Hepcidin expression is stipulated to contribute to the underlying mechanism linking the effect of vitamin D supplements on improvement of iron status

12. Honorarium and incentives to respondents :

Participants will be given RM50 cash upon completion of the study. However, the participants will not be reimbursed with any other expenses (including travelling and participation time) incurred during participation in the study.

13. References :

Ahmed, F., Coyne, T., Dobson, A., & McClintock, C. (2008). Iron status among Australian adults: findings of a population based study in Queensland, Australia. Asia Pacific Journal of Clinical Nutrition, 17(1), 40-47.

Bacchetta, J., Zaritsky, J. J., Sea, J. L., Chun, R. F., Lisse, T. S., Zavala, K., . . . Hewison, M. (2013). Suppression of iron-regulatory hepcidin by vitamin D. Journal of the American Society of Nephrology, 25, 564-572. doi:10.1681/asn.2013040355

Casgrain, A., Collings, R., Harvey, L. J., Hooper, L., & Fairweather-Tait, S. J. (2012). Effect of iron intake on iron status: a systematic review and meta-analysis of randomised controlled trials. The American Journal of Clinical Nutrition, 96(4), 768-780.

Detert, H., Hedlund, S., Anderson, C. D., Rodvall, Y., Festin, K., Whiteman, D. C., & Falk, M. (2015). Validation of sun exposure and protection index (SEPI) for estimation of sun habits. Cancer Epidemiology, 39(6), 986-993. doi:https://doi.org/10.1016/j.canep.2015.10.022

Ernst, J. B., Tomaschitz, A., Grübler, M. R., Gaksch, M., Kienreich, K., Verheyen, N., . . . Zittermann, A. (2016). Vitamin D supplementation and hemoglobin levels in hypertensive patients: a randomized controlled trial. International Journal of Endocrinology, 2016. doi:dx.doi.org/10.1155%2F2016%2F6836402

Ferreira da Silva, L., Dutra-de-Oliveira, J. E., & Marchini, J. S. (2004). Serum iron analysis of adults receiving three different iron compounds. Nutrition Research, 24(8), 603-611. doi:10.1016/j.nutres.2003.10.013

Ganz, T., Olbina, G., Girelli, D., Nemeth, E., & Westerman, M. (2008). Immunoassay for human serum hepcidin. Blood, 112(10), 4292-4297. doi:10.1182/blood-2008-02-139915

Gera, T., Sachdev, H. S., & Boy, E. (2012). Effect of iron-fortified foods on hematologic and biological outcomes: systematic review of randomized controlled trials. The American Journal of Clinical Nutrition, 96(2), 309-324. doi:10.3945/ajcn.111.031500

Han, S. S., Kim, M., Kim, H., Lee, S. M., Oh, Y. J., Lee, J. P., . . . Kim, Y. S. (2013). Non-linear relationship between serum 25-hydroxyvitamin D and hemoglobin in Korean females: The Korean National Health and Nutrition Examination Survey 2010–2011. PLoS One, 8(8), e72605.

Haniff, J., Zaher, Z. M. M., Das, A., Onn, L. T., Sun, C. W., Nordin, N. M., . . . Malaysian Anemia Study, G. (2007). Anemia in pregnancy in Malaysia: a cross-sectional survey. Asia Pacific Journal of Clinical Nutrition, 16(3), 527-536.

Hansen, L., Tjønneland, A., Køster, B., Brot, C., Andersen, R., Lundqvist, M., . . . Olsen, A. (2016). Sun Exposure Guidelines and Serum Vitamin D Status in Denmark: The StatusD Study. Nutrients, 8(5), 266. doi:10.3390/nu8050266

Holick, M. F., Binkley, N. C., Bischoff-Ferrari, H. A., Gordon, C. M., Hanley, D. A., Heaney, R. P., . . . Weaver, C. M. (2011). Evaluation, treatment, and prevention of vitamin D deficiency: an Endocrine Society clinical practice guideline. The Journal of Clinical Endocrinology & Metabolism, 96(7), 1911-1930.

Kaushansky, K., Lichtman, M., Prchal, J., Levi, M., Press, O., Burns, L., & Caligiuri, M. (2015). Williams Haematology (9th ed.). New York, USA: McGraw Hill Education.

Lee, R. D., & Nieman, D. C. (2010). Nutritional Assessment (5 ed.). New York, USA: McGraw-Hill Education.

Loh, S., & Khor, G. (2010). Iron intake and iron deficiency anaemia among young women in Kuala Lumpur. Malaysian journal of nutrition and Health Sciences, 6(1), 63-70.

Lynch, S. R. (2011). Why nutritional iron deficiency persists as a worldwide problem. The Journal of Nutrition, 141(4), 763S-768S.

McDonnell, S. M., & Witte, D. (1997). Hereditary hemochromatosis: preventing chronic effects of this underdiagnosed disorder. Postgraduate medicine, 102(6), 83-94.

McGillivray, G., Skull, S. A., Davie, G., Kofoed, S. E., Frydenberg, A., Rice, J., . . . Carapetis, J. R. (2007). High prevalence of asymptomatic vitamin D and iron deficiency in East African immigrant children and adolescents living in a temperate climate. Archives of Disease in Childhood, 92(12), 1088-1093.

Pasricha, S.-R., Drakesmith, H., Black, J., Hipgrave, D., & Biggs, B.-A. (2013). Control of iron deficiency anemia in low- and middle-income countries. Blood, 121(14), 2607.

Riccio, E., Sabbatini, M., Bruzzese, D., Capuano, I., Migliaccio, S., Andreucci, M., & Pisani, A. (2015). Effect of paricalcitol vs calcitriol on hemoglobin levels in chronic kidney disease patients: a randomized trial. PLoS One, 10(3), e0118174.

Ross, A. C., Taylor, C. L., Yaktine, A. L., & Del Valle, H. B. (2011). Dietary Reference Intakes for calcium and vitamin D: Food and Nutrition Board. Washington DC, USA: Institute of Medicine of the National Academies Press.

SACN. (2016). Vitamin D and Health. London, United Kingdom: TSO.

Sharma, S., Jain, R., & Dabla, P. K. (2015). The role of 25-hydroxy vitamin D deficiency in iron deficient children of North

India. Indian Journal of Clinical Biochemistry, 30(3), 313-317.
Sim, J. J., Lac, P. T., Liu, I. L. A., Meguerditchian, S. O., Kumar, V. A., Kujubu, D. A., & Rasgon, S. A. (2010). Vitamin D deficiency and anemia: a cross-sectional study. Annals of Hematology, 89(5), 447-452.
Smith, E. M., Alvarez, J. A., Kearns, M. D., Hao, L., Sloan, J. H., Konrad, R. J., . . . Tangpricha, V. (2016). High-dose vitamin D3 reduces circulating hepcidin concentrations: A pilot, randomized, double-blind, placebo-controlled trial in healthy adults. Clinical Nutrition. doi:dx.doi.org/10.1016/j.clnu.2016.06.015
Smith, E. M., & Tangpricha, V. (2015). Vitamin D and Anemia: Insights into an Emerging Association. Current opinion in endocrinology, diabetes, and obesity, 22(6), 432-438. doi:10.1097/MED.0000000000000199
Souza, A. I. d., Batista Filho, M., Bresani, C. C., Ferreira, L. O. C., & Figueiroa, J. N. (2009). Adherence and side effects of three ferrous sulfate treatment regimens on anemic pregnant women in clinical trials. Cadernos de Saúde Pública, 25(6), 1225-1233.
Tee, E. S., Ismail, M. N., Nasir, A., & Katijah, I. (1997). Nutrient Composition of Malaysian Foods. National Subcomittee on Protein: Food Habits Research and Development Malaysia. Retrieved from
Vieth, R. (2005). Chapter 61: The pharmacology of vitamin D, including fortification strategies. In D. Feldman & F. Glorieux (Eds.), Vitamin D (Vol. 2, pp. 995-1015): Elsevier Acad Press.
WHO. (1998). Obesity: preventing and managing the global epidemic : report of a WHO consultation on obesity. Geneva, Switzerland: World Health Organization.
WHO. (2008). Worldwide prevalence of anemia 1993-2005: WHO database on anemia. Geneva, Switzerland: WHO Press, World Health Organization.
WHO. (2015). The global prevalence of anaemia in 2011. Geneva, Switzerland: World Health Organisation.
WHO/CDC. (2004). Assesing the iron status of populations : including literature reviews : report of a Joint World Health Organization/Centers for Disease Control and Prevention Technical Consultation on the Assessment of Iron Status at the Population Level. Geneva, Switzerland: World Health Organization/Centers for Disease Control and Prevention.
WHO/UNICEF/UNU. (2001). Iron deficiency anemia : assessment, prevention and control. A guide for programme managers. . Geneva, Switzerland: World Health Organization.
Yoon, J. W., Kim, S. W., Yoo, E. G., & Kim, M. K. (2012). Prevalence and risk factors for vitamin D deficiency in children with iron deficiency anemia. Korean journal of pediatrics, 55(6), 206-211.
Zughaier, S. M., Alvarez, J. A., Sloan, J. H., Konrad, R. J., & Tangpricha, V. (2014). The role of vitamin D in regulating the iron-hepcidin-ferroportin axis in monocytes. Journal of Clinical & Translational Endocrinology, 1(1), e19-e25.

APPENDICES :

Appendix I: Flow Chart of Study Design

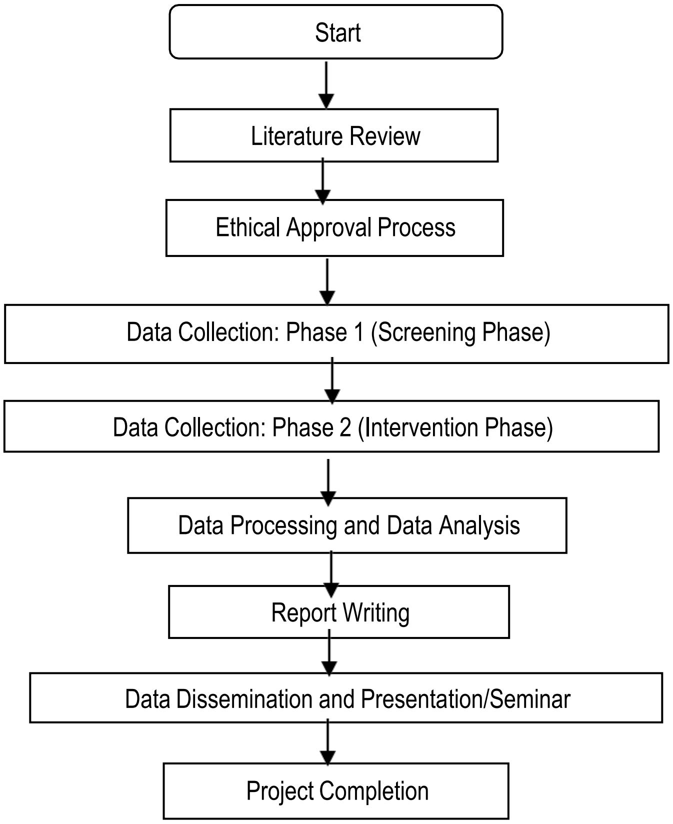

Figure 9.1 Study Design

Appendix II: Flow Chart of Study Protocol

Study population:
Childbearing-aged women (19-40 years)

↓

[a]Screening Phase (n=~*736*):
- [b]Marginally low iron stores based on plasma ferritin threshold of < 20 µg/l
- Non anaemic based on haemoglobin threshold of > 12 g/dl

↓

Randomisation [c](n=120)

Vitamin D3 (4000 IU)<br>(n=60)<br>8 weeks	Placebo<br>(n=60)<br>8 weeks

↓

<u>Baseline measurements (Clinic 1 - Week 0):</u>

- Anthropometric measurements of height & weight using wall mounted digital stadiometer (Model 264 SECA, Hamburg, Germany) and electronic scale (Model 875 SECA, Hamburg, Germany) respectively.
- Collection of first 3-day food diary to estimate their habitual dietary intake and to account for potential confounding from dietary intake. Dietary data will be analysed using Nutritionist Pro Software (Axxya System LLC, Washington, USA) and compared to the Malaysian Recommended Nutrient Intake (RNI 2017).
- Sun exposure quantification using a set of validated and modified questionnaire (Detert et al., 2015; Hansen et al., 2016)
- 10 ml fasted venous blood sample to determine iron and vitamin D status which includes full blood counts, ferritin, C-reactive protein, hepcidin, 25(OH)D, parathyroid hormones and other iron/vitamin D biomarkers concentrations

↓

<u>Interim measurements (Clinic 2 - Week 4):</u>

- Anthropometric measurements (weight)
- Administration of post-intervention 3-day food diary
- Sun exposure quantification
- 10 ml fasted venous blood sample

↓

<u>Post-intervention measurements (Clinic 3 - Week 8):</u>

- Anthropometric measurements (weight)
- Collection of post-intervention 3-day food diary
- Sun exposure quantification
- 10 ml fasted venous blood sample

Figure 9.2 Clinical Study Protocol

CLINICAL STUDY PROTOCOL

Document submitted for ethical approval from JKEUPM 2020

1. Research Title :	Effect of vitamin D3-fortified fruit juice supplementation on the recovery of iron status in childbearing-aged women with marginally low iron stores
2. Principal Investigator :	Dr. Salma Faeza Binti Ahmad Fuzi
3. Co- Investigators :	1. Assoc. Prof. Dr Loh Su Peng<br>2. Madam Norhafizah Ab Manan<br>3. Prof. Dr Muhammad Najib Mohamad Alwi

4. Research questions/objectives and hypotheses :

Therefore, the present study is designed to utilise vitamin D3-fortified fruit juice as supplements that may potentially act as an iron absorption enhancer to improve iron status in the Malaysian child-bearing aged women with low iron stores. In addition to investigating the efficacy, this study is also designed to assess the effect of a higher dose of vitamin D3 in the fortified fruit juice (4000 IU) on iron metabolism. The study will include the measurement of plasma hepcidin and 25(OH)D concentrations to investigate a possible mechanism that links vitamin D and iron deficiency, as postulated from the existing literature. For that reason, the aim of the study was to investigate the effect of an 8-week vitamin D3-fortified fruit juice supplementation on iron status indicators, including hepcidin concentration in childbearing aged Malaysian women with marginal iron stores. It is hypothesised that there will be a significant improvement in haematological indicators following 8-week daily vitamin D3- fortified fruit juice supplementation in the vitamin D group compared to placebo group. It is also hypothesised that plasma hepcidin concentration will be reduced following 8-week daily vitamin D3-fortified fruit juice supplementation, which results in increased iron stores.

5. Clinical Study Protocol :

5.1 Study Design

The study is a placebo controlled, double-blind randomised controlled trial, designed to investigate the effect of an 8-week vitamin D3-fortified fruit juice supplementation on haematological indicators and hepcidin response in a cohort of marginally-low iron stores Malaysia childbearing-aged women. The study will be registered with the NMMR and at clinicaltrials.gov and ethical approval for the study protocol will be obtained from the Ethic Committee for Research Involving Human Subject UPM (JKEUPM) (Figure 5.1) (Appendix I).

The study will be carried out for the duration of 8 weeks in Selangor, Malaysia. The data will be collected between June 2020 and June 2021. The inclusion criteria will be women of child bearing age, healthy, aged 19-40 years, and non-pregnant nor lactating. The women will be excluded if they had a history of gastrointestinal disorder (celiac disease, Crohn's disease, irritable bowel syndrome gastroesophageal reflux disease, peptic ulcers and other related gastrointestinal disorders which may cause nutrient malabsorption) and iron metabolic disorders such as iron overload, had donated blood since the past 6 months, and regularly consuming nutritional supplements (iron, vitamin D, vitamin C, calcium).

Figure 5.2 outlines the clinical study protocol which included 2 main phases of the study (Appendix II). Phase 1 will be the recruitment and screening phase when potential participants will be screened for marginally low iron stores, identified and randomised. Phase 2 will be the intervention phase where all the eligible participants will be required to consume either vitamin D3-fortified fruit juice containing 4000 IU (100 mcg) (vitamin D group) or placebo-fruit juice (placebo group) daily for the duration of 8 weeks.

5.1.1 Phase 1: Screening

All participants will be provided with a participant information sheet (PIS) and a date will be arranged for the venous blood collection to ascertain the plasma ferritin and haemoglobin concentrations, which will be used to indicate iron stores and haemoglobin levels in the present study. The potential participants will be given a written informed consent and will be asked to complete a screening questionnaire to ensure eligibility criteria is met. The screening questionnaire will be used to obtain socio-demographic characteristics including age, ethnicity, religion, educational level and household income. During the screening clinic session, 1 ml of venous blood will be collected at the clinical laboratory within the Department of Nutrition & Dietetics at the UPM by the researcher who is a trained phlebotomist. The whole blood and plasma sample collected will be used to determine the concentrations of plasma ferritin and haemoglobin. Based on the blood analysis, participants will be notified of their results via email or phone. The eligible participant who has marginally low iron stores (based on plasma ferritin concentration of between < 20 ug/l) and non-anaemic (based on haemoglobin concentration of > 12 g/dl or higher) will be then invited to continue to Phase 2 of the study. Participants who had abnormally high plasma ferritin concentrations (> 150 ug/l) and low haemoglobin concentrations (< 12 g/dl) from the screening will be advised to arrange a check-up with their registered general practitioner and not included in the study. An initial testing of fruit juices will be carried out during the baseline clinic session for participants to test the palatability of the fruit juices.

5.1.2 Phase 2: Intervention

A total of 120 participants who are eligible to continue with the study will be randomised to receive either 4000 IU (100 mcg) of vitamin D3-fortified fruit juice or placebo-fruit juice. The participants will be instructed to consume the vitamin D3-fortified fruit juice or placebo-fruit juice in the morning by diluting the powder in 150 ml of water, daily, for the 8-week duration of the study. All the participants will be reminded to not alter their dietary habits and physical activity, in addition, to abstaining from donating blood during the course of the study which may interfere with interpretation of study findings.

5.1.3 Study clinic sessions

Participants will all be required to attend clinic sessions after overnight fasts of approximately 8 hours and will be expected to attend 3 clinics in total for the duration of study. The clinic sessions will be in the clinical laboratory within the Department of Nutrition & Dietetics at the UPM where the initial screening session carried out. Participants will be asked to consume only water during the overnight fast. Each clinic will last approximately 20 minutes and will take place between 8-10 am in the

morning.

The details of each clinic sessions are as follows:

Clinic 1 (Week 0, baseline): Height (cm) will be measured by using digital stadiometer and weight (kg) using weighing scales, followed by collection of a 10 ml fasted venous blood sample collection. Participants will be provided with a 3-day food diary to be completed at home before the clinic and collected during the clinic. An 8-week supply of a supplement pot, containing fruit juice powder in sachets with assigned participant ID will be provided to the participants. The protocol will be explained to participants and an email will be sent as a reminder to the participants one day before the following clinic.

Clinic 2 (Week 4, interim): The same procedure will be repeated except for measurement of height. Participants will be then be given a further 3-day food diary to be completed before the next clinic.

Clinic 3 (Week 8, post-intervention): The same procedure will be repeated except for measurement of height. The final 3-day food diary and supplement pot (with any) of the remaining fruit juice powder in sachets to calculate the compliance will be collected.

5.2 Randomisation and blinding of participants

5.2.1 Randomisation

The randomisation process will be carried out using a computer-generated software (www.randomization.com) by a third party, independent to the study. Participants (n=120) will be randomised to 2 groups: vitamin D3-fortified fruit juice (vitamin D group) or placebo-fruit juice (placebo group).

The third party will allocate 62 D3-fortified fruit juice sachets and placebo-fruit juice sachets into each supplement pot according to the generated plan. An excess of 6 sachets in each pot will enable the researcher to estimate compliance once the study is completed. Each supplement pot will then be sealed in a tamper proof supplement pot and numbered ready to be provided to participants.

5.2.2 Blinding

The participants and researcher will be double-blinded as to which groups participants will be assigned. The researcher will administer the numbered supplement pot (i.e; 001) to the participants based on the sequence that participants attend their baseline clinic (Week 0). The blinding will be maintained throughout the study period of 8 weeks and allocation will not be unlocked until the end of the data analysis or during any adverse event.

5.3 Adverse effect and safety

The vitamin D3-fortified fruit juice powder supplements will be packaged in individual sachet for daily consumption and produced specific to research requirement and in accordance to Good Manufacturing Practice (GMP) standards. For safety

purposes, powder produced will be sent for microbiological tests and heavy metals for it to be deemed safe for human consumption. These tests will be carried out on the powder before it is packaged into sachets and in the finished products.

The vitamin D recommended intake in different populations worldwide is currently set to between 100-800 IU (Adams & Hewison, 2010), and there is no international consensus on the optimal dietary intake of vitamin D (Brouwer-Brolsma et al., 2013). The range between 100-800 IU is regarded as underestimated, contributes to the difficulty in maintaining sufficient vitamin D status, hence, the need for additional vitamin D from supplement form (Adams & Hewison, 2010). The RNI proposed includes the daily intake from either naturally-contained vitamin D food sources, vitamin D-fortified foods or supplements. Table 9.1 summarises the recommended dietary and supplement intakes from various sources worldwide.

Table 9.1 Overview of vitamin D dietary and supplement recommendations for adults

Reference	Dietary	Supplements (D2 or D3)
UK SACN<br>(SACN, 2016)	400 IU/day (10 µg)	400 IU/day (10 µg)
IOM<br>(Ross et al., 2011)	600 IU/day (15 µg)	600 IU/day (15 µg)
The Endocrine Society<br>(Holick et al., 2011)	600 IU/day (15 µg)	6000 IU/day (150 µg) or 50000 IU/week (1250 µg) (8 weeks)
Vitamin D Council	5000 IU/day (125 µg)	5000 IU/day (125 µg)
Vieth (2005)	200 IU/day (5 µg)	4000 IU/day (100 µg)

The dose of 4000 used in the study is a safe dose for adults and not associated with any adverse effect. Various institutions recommended different safe dosages between 400-6000 IU/day or up to 50000 IU/week (Holick et al., 2011; Ross, Taylor, Yaktine, & Del Valle, 2011; Vieth, 2005). Published studies investigating the use of vitamin D supplements or fortified food products on iron and vitamin D status biomarkers have not reported adverse events (Appendix III).

5.4 Honorarium and incentives to participants

Participants will be given RM50 cash upon completion of the study. However, the participants will not be reimbursed with any other expenses (including travelling and participation time) incurred during participation in the study.

APPENDICES :

Appendix I: Flow Chart of Study Design

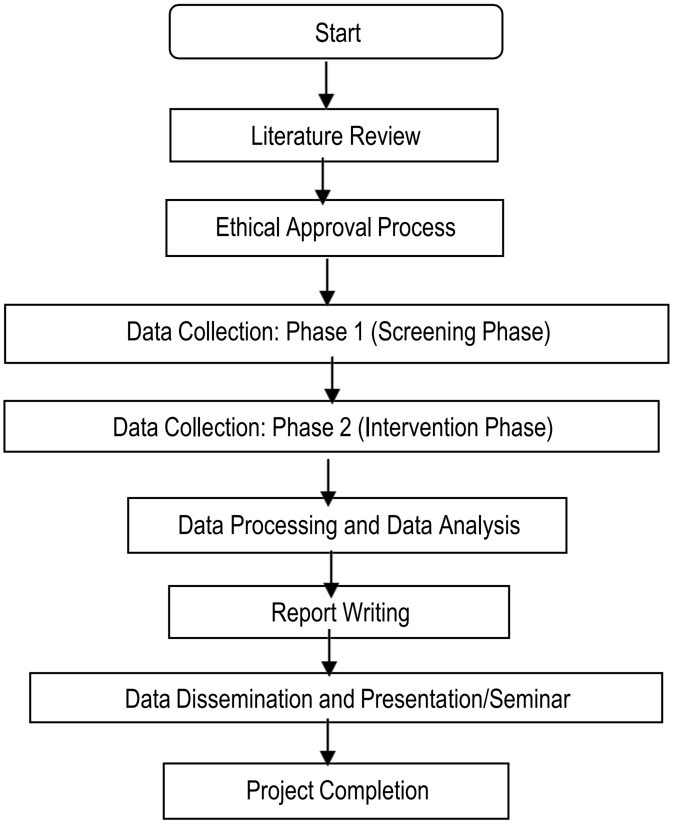

Figure 5.1 Study Design

Appendix II: Flow Chart of Clinical Study Protocol

Study population:
Childbearing-aged women (19-40 years)

↓

[a]Screening Phase (n=~736):
- [b]Marginally low iron stores based on plasma ferritin threshold of < 20 µg/l
- Non anaemic based on haemoglobin threshold of > 12 g/dl

↓

Randomisation [c](n=120)

Vitamin D3 (4000 IU)<br>(n=60)<br>8 weeks	Placebo<br>(n=60)<br>8 weeks

↓

Baseline measurements (Clinic 1 - Week 0):

- Anthropometric measurements of height & weight using wall mounted digital stadiometer (Model 264 SECA, Hamburg, Germany) and electronic scale (Model 875 SECA, Hamburg, Germany) respectively.
- Collection of first 3-day food diary to estimate their habitual dietary intake and to account for potential confounding from dietary intake. Dietary data will be analysed using Nutritionist Pro Software (Axxya System LLC, Washington, USA) and compared to the Malaysian Recommended Nutrient Intake (RNI 2017).
- Sun exposure quantification using a set of validated and modified questionnaire (Detert et al., 2015; Hansen et al., 2016)
- 10 ml fasted venous blood sample to determine iron and vitamin D status which includes full blood counts, ferritin, C-reactive protein, hepcidin, 25(OH)D, parathyroid hormones and other iron/vitamin D biomarkers concentrations

↓

Interim measurements (Clinic 2 - Week 4):

- Anthropometric measurements (weight)
- Administration of post-intervention 3-day food diary
- Sun exposure quantification
- 10 ml fasted venous blood sample

↓

Post-intervention measurements (Clinic 3 - Week 8):

- Anthropometric measurements (weight)
- Collection of post-intervention 3-day food diary
- Sun exposure quantification
- 10 ml fasted venous blood sample

Figure 5.2 Clinical Study Protocol

[a]The prevalence of iron deficiency based on plasma ferritin concentration of < 15 µg/l in women aged 18-40 years in Malaysia was reported to be approximately 33% (Loh & Khor, 2010). The prevalence of adult women who consumed vitamin and mineral supplements (VMS) based on the Malaysian Nutrition Adult Survey (MANS) 2014 was reported to be approximately 32.1% (Mohd Zaki et al., 2018).

To take into account the combination of both data, approximately 736 women would have to be screened to achieve the required sample size.

[b]The potential participants will be then screened to determine their plasma ferritin and haemoglobin concentrations to include women with marginal low iron stores [plasma ferritin concentration < 20 µg/l] (Ahmed, Coyne, Dobson, & McClintock, 2008; McDonnell & Witte, 1997) and non-anaemic [haemoglobin concentration < 12 g/dl] (WHO/UNICEF/UNU, 2001).

[c]Sample size was estimated by using (mean ± S.D) serum ferritin concentrations (ug/dl) from a randomised trial carried out by Blanco Rojo et al. (2011) in 41 women aged 18-35 years with low iron stores to determine the effect of iron-fortified fruit juice consumption on bothiron and vitamin D status. At week 8 of study, it was observed that (mean ± S.D) serum ferritin concentrations were significantly higher in the vitamin D group (34.1 ± 14.8 ug/l), compared to 24.8 ± 17.7 ug/l in the placebo group ($p<0.05$). Based on this study, the effect size is calculated by dividing the mean difference (9.3 ng/ml) by the pooled standard deviation (16.29). With an effect size of 0.571, the total sample size will be required in the present study is 50/group (Power=0.80, α err prob=0.05). Allowing for a 20% drop-out rate, the total sample size required to demonstrate a significant difference in serum ferritin concentrations between vitamin D group and placebo is estimated to be 120 (60 participants/group).
